# Preliminary Anticonvulsant and Toxicity Screening of Substituted Benzylidenehydrazinyl-*N*-(6-substituted benzo*[d]*thiazol-2-yl)propanamides

**DOI:** 10.1155/2014/194652

**Published:** 2014-12-11

**Authors:** Ruhi Ali, Nadeem Siddiqui

**Affiliations:** Department of Pharmaceutical Chemistry, Faculty of Pharmacy, Jamia Hamdard, New Delhi 1100062, India

## Abstract

Keeping in view the structural requirements suggested in the pharmacophore model for anticonvulsant activity, a new series of 3-(2-(substitutedbenzylidene)hydrazinyl)-*N*-(substituted benzo*[d]*thiazol-2-yl)-propanamides were synthesized with aromatic hydrophobic aryl ring (A), NH–C=O as hydrogen bonding domain (HBD), nitrogen atom as electron donor (D), and phenyl as distal aryl ring (C). Synthesized compounds were characterized by FTIR, ^1^H NMR, ^13^C NMR, mass spectroscopy, and elemental analysis. Preliminary *in vivo* anticonvulsant screening (phase I) was performed by two most adopted seizure models, maximal electroshock seizure (MES) and subcutaneous pentylenetetrazole (scPTZ). Based on anticonvulsant screening results, two compounds, **5h** and **5p**, were found to be most active; they exhibited activity comparable to standard drugs phenytoin (PHY) and carbamazepine (CBZ). These active compounds were subjected to phase II and phase III screening, where they displayed much higher protective index (PI) in comparison to the standard drugs. In phase IV screening, the bioavailability of active compounds was assessed on oral administration. Further, preliminary safety profiles of **5h** and **5p** were evaluated by the neurotoxicity testing and liver enzyme estimation.

## 1. Introduction

Epilepsy is one of the most prevalent noncommunicable neurological conditions. It is a main cause of disability and mortality [1] and characterized by paroxysmal, excessive, and hyperasynchronous discharges of large numbers of neurons [2]. More than 10 million people in India are afflicted with epilepsy [3]. The prevalence of epilepsy is higher in the rural (1.9%) as compared with the urban population (0.6%) [4, 5]. Every year about 2.4 million new cases are added to these figures [6, 7]. Several new anticonvulsants like vigabatrin, lamotrigine, gabapentin, topiramate, felbamate, rufinamide, and levetiracetam have been recently introduced in clinical practices. Regardless of the introduction of these new drugs in the past decade, up to one-third of epilepsy patients developed resistance to optimum drug treatment [8]. The therapeutic efficiency of these well-known established drugs in reducing seizure is prevailed over by some detrimental side effects such as headache, nausea, hepatotoxicity, gastrointestinal disturbances, and hirsutism [9, 10]. These facts triggered the further scope and search for newer more effective and less toxic anticonvulsants.

Benzothiazole scaffold is amongst the commonly occurring heterocyclic nuclei in many marine as well as natural plant products. It is a promising bicyclic ring system with multiple biological applications [11–15]. In recent years, extensive research has focused on developing novel benzothiazole derivatives to improve anticonvulsant activities.

In view of these facts and as a part of our continuing studies in the area of anticonvulsant agents, it was thought of interest to synthesize some newer derivatives of benzothiazole as anticonvulsant agents. A pharmacophore model along with physicochemical determination provides a useful tool for designing prototypic molecules and explanation of probable interactions. In terms of interaction at binding site, the titled compounds have common structural features such as aromatic hydrophobic aryl ring (A), NH–C=O as hydrogen bonding domain (HBD), nitrogen atom as electron donor (D), and phenyl as distal aryl ring (C) [16]. In the present study, therefore, we hereby describe the synthesis and preliminary anticonvulsant evaluation of some new 3-[2-(substituted benzylidene)hydrazinyl]-N-(substituted benzo*[d]*thiazol-2yl)-propanamides.

## 2. Experimental

### 2.1. Measurements

The entire chemicals used in the synthesis were procured from E. Merck and S. D. Fine Chemicals. A Thin layer chromatography (TLC) was performed with Silica gel 60 F254 TLC aluminium sheet (Merck) using toluene : ethyl acetate : formic acid (5 : 4 : 1) and benzene : acetone (9 : 1) as eluents. Ashless Whatmann number 1 filter paper was used for vacuum filtration. Melting points were determined by using open capillary tubes in a Hicon melting point apparatus (Hicon, India) and are uncorrected. The purity of the compounds was confirmed through elemental analysis. The elemental analyses (C, H, N, and S) of all compounds were performed on the CHNS Elimentar (Analysen systime, GmbH) Germany Vario EL III and results were within ±0.4% of the theoretical values. Fourier transform infrared (FT-IR) spectra were recorded in KBr pellets on a Shimadzu FT-IR spectrometer. ^1^HNMR and ^13^CNMR spectra in DMSO-*d*_*6*_/CDCl_3_ solutions were, respectively, recorded at 400 and 100 MHz with Bruker 400 Ultrashield TM NMR spectrometer using TMS [(CH_3_)_4_Si] as internal standard. Splitting patterns are nominated as follows: s, singlet; bs, broad singlet; d, doublet; t, triplet; m, multiplet. The NH protons were D_2_O exchanged for their spectral characterization. The mass spectra were recorded using Waters Micromass ZQ 2000 Spectrophotometer (Jamia Hamdard, New Delhi, India).

#### 2.1.1. Synthesis of 3-[2-(2-Substituted benzylidene)hydrazinyl]-*N*-(6-substitutedbenzo*[d]*thiazol-2-yl)propanamides (**5a**–**t**)

*Step I: 6-Substituted-1, 3-benzothiazole-2-amines (* ***1a*** *–* ***d*** *).* A mixture of substituted aniline (0.01 mol) and potassium thiocyanate (0.01 mol) in glacial acetic acid (10%) was cooled and stirred. Bromine (0.01 mol) was added dropwise to this solution at such a rate to keep the temperature below 10°C throughout the addition. For additional 3 h, stirring was continued and the separated hydrochloride salt was filtered, washed with acetic acid, and dried. Reaction mixture was dissolved in hot water and neutralized with aqueous ammonia solution (25%), filtered, washed with water, dried, and recrystallized with benzene to obtain 6-substituted-1, 3-benzothiazole-2-amines (**1a**–**d**).

*Step II: Synthesis of N-(6-Substituted benzo[d]thiazol-2-yl)propanamides (* ***2a*** *–* ***d*** *).* To the solution of substituted-1, 3-benzothiazole-2-amines (**1a**–**d**, 0.1 mol) in DMF, propionyl chloride (0.2 mol) was added slowly with continuous stirring. The reaction mixture was stirred for 12 hrs. On cooling,* N*-(6-substituted benzo*[d]*thiazol-2-yl)propanamides (**2a**–**d**) was obtained.

*Step III: 3-Bromo-N-(6-substituted benzo[d]thiazol-2-yl)propanamides (* ***3a*** *–* ***d*** *).* Bromine in glacial acetic acid (10 mL) was added dropwise to the solution of* N*-(6-substituted benzo*[d]*thiazol-2-yl)propanamides in DMF at 0°C. Stirring was continued for 24 hrs. Mixture of compounds was obtained which was separated by column chromatography to yield 3-bromo-*N*-(6-substituted benzo*[d]*thiazol-2-yl)propanamides (**3a**–**d**).

*Step IV: N-(6-Substituted benzo[d]-thiazol-2yl)-3-hydrazinylpropanamides (* ***4a*** *–* ***d*** *).* Compound** 3a** (0.1 mol) and hydrazine hydrate (0.3 mol) in ethanol (50 mL) were refluxed for 2 h. The excess of solvent was removed under reduced pressure and recrystallized from chloroform-hexane (3 : 1) to yield crystals of compound** 4a**. All other compounds of the series (**4b**–**d**) were also prepared by the above specified procedure with slight variation in reaction time.

*Step V: 3-[2-(2-Substituted benzylidene)hydrazinyl]-N-(6-substituted benzo[d]thiazol-2-yl)propanamides (* ***5a*** *–* ***t*** *).* The solution of compound** 4a** in glacial acetic acid (5 mL) and ethanol (10 mL) was heated to boiling and refluxed with benzaldehyde (0.12 mol) for 5 h. The refluxed solution was cooled to room temperature and kept overnight. The solid (**5a**) was collected out, washed with methanol, dried, and recrystallized from methanol to get the pure compound. All other compounds of the series (**5b**–**t**) were also prepared by using respective aromatic aldehydes by the above specified procedure with slight variation in reaction time.

*3-(2-Benzylidenehydrazinyl)-N-(6-chlorobenzo[d]thiazol-2-yl)propanamides (* ***5a*** *).* Yield 65%; IR (KBr) *ν* cm^−1^: 3316 (N–H str.), 1644 (C=O), 1610 (CH=N); ^1^H NMR (DMSO-*d*_6_) *δ* ppm: 2.50 (t, 2H, CH_2_C=O), 2.98 (t, 2H,  CH_2_NH), 5.86 (br s, 1H, NH, D_2_O exchangeable), 7.40–7.80 (m, 5H, Ar–H), 7.50–8.19 (m, 3H, Benzothiazole-H), 8.40 (s, 1H, CH=N), 9.20 (s, 1H, CONH, D_2_O exchangeable); ^13^C NMR (CDCl_3_) *δ* ppm: 32.9, 46.2, 118.1, 121.3, 125.9, 128.9, 129.7, 131.1, 132.6, 133.2, 143.9, 151.9, 173.1, 174.5; MS (70 ev), *m*/*z*: 358.09 [M+H]^+^; Anal. Calcd. for C_17_H_15_ClN_4_OS: C, 56.90, H, 4.21, N, 15.61, S, 8.74. Found: C, 56.88, H, 4.19, N, 15.58, S, 8.70.

*3-(2-Benzylidenehydrazinyl)-N-(6-flourobenzo[d]thiazol-2-yl)propanamides (* ***5b*** *).* Yield 62%; IR (KBr) *ν* cm^−1^: 3320 (N–H str.), 1640 (C=O), 1569 (CH=N); ^1^H NMR (DMSO-*d*_6_) *δ* ppm: 2.45 (t, 2H, CH_2_), 2.95 (t, 2H,  CH_2_NH), 6.10 (br s, 1H, NH, D_2_O exchangeable), 7.42–7.58 (m, 5H, Ar–H), 7.46–8.20 (m, 3H, Benzothiazole-H), 10.02 (s, 1H, CONH, D_2_O exchangeable), 8.36 (s, 1H, CH=N); ^13^C NMR (CDCl_3_) *δ* ppm: 32.7, 46.0, 121.8, 123.7, 126.0, 129.1, 129.9, 131.7, 134.8, 147.9, 144.9, 173.1, 174.9, MS (70 ev), *m*/*z*: 342.10 [M+H]^+^; Anal. Calcd. for C_17_H_15_FN_4_OS: C, 59.63, H, 4.45, N, 16.36, S, 9.33. Found: C, 59.65, H, 4.49, N, 16.40, S, 9.29.

*3-(2-Benzylidenehydrazinyl)-N-(6-methylbenzo[d]thiazol-2-yl)propanamides (* ***5c*** *).* Yield 68%; IR (KBr) *ν* cm^−1^: 3325 (N–H str.), 1680 (C=O), 1580 (CH=N); ^1^H NMR (DMSO-*d*_6_) *δ* ppm: 2.32 (s, 3H, CH_3_), 2.60 (t, 2H, CH_2_), 2.93 (t, 2H,  CH_2_NH), 5.89 (br s, 1H, NH, D_2_O exchangeable), 7.16–7.58 (m, 5H, Ar–H), 7.56–8.10 (m, 3H, Benzothiazole-H), 8.40 (s, 1H, CH=N), 10.02 (s, 1H, CONH, D_2_O exchangeable); ^13^C NMR (CDCl_3_) *δ* ppm: 24.0, 32.9, 46.2, 121.5, 121.7, 124.7, 126.0, 128.9, 129.2, 131.7, 134.8, 143.9, 146.9, 173.7, 174.5; MS (70 ev), *m*/*z*: 338.10 [M+H]^+^; Anal. Calcd. for C_18_H_18_N_4_OS: C, 63.80, H, 5.54, N, 16.68, S, 9.47. Found: C, 63.78, H, 5.56, N, 16.70, S, 9.45.

*3-(2-Benzylidenehydrazinyl)-N-(6-methoxybenzo[d]thiazol-2-yl)propanamides (* ***5d*** *).* Yield 73%; IR (KBr) *ν* cm^−1^: 3350 (N–H str.), 1665 (C=O), 1601 (CH=N); ^1^H NMR (DMSO-*d*_6_) *δ* ppm: 2.66 (t, 2H, CH_2_), 2.90 (t, 2H,  CH_2_NH), 3.70 (s, 3H, OCH_3_), 5.90 (br s, 1H, NH, D_2_O exchangeable), 7.26–7.59 (m, 5H, Ar–H), 7.42–8.17 (m, 3H, Benzothiazole-H), 8.20 (s, 1H, CH=N), 10.30 (s, 1H, CONH, D_2_O exchangeable); ^13^C NMR (CDCl_3_) *δ* ppm: 32.8, 46.2, 55.9, 105.0, 113.2, 122.5, 125.7, 128.9, 129.2, 131.1, 133.8, 141.9, 143.9, 173.7, 174.5; MS (70 ev), *m*/*z*: 354.01 [M+H]^+^; Anal. Calcd. for C_18_H_18_N_4_O_2_S: C, 61.10, H, 5.12, N, 15.79, S, 8.98. Found: C, 61.17, H, 5.10, N, 15.76, S, 8.99.

*N-(6-Chlorobenzo[d]thiazol-2-yl)-3-[2-(2-hydroxybenzylidene)hydrazinyl]-propanamides (* ***5e*** *).* Yield 63%; IR (KBr) *ν* cm^−1^: 3350 (N–H str.), 1665 (C=O), 1609 (CH=N); ^1^H NMR (DMSO-*d*_6_) *δ* ppm: 2.63 (t, 2H, CH_2_), 2.91 (t, 2H,  CH_2_NH), 5.40 (s, 1H, OH), 6.12 (br s, 1H, NH, D_2_O exchangeable), 6.80–7.40 (m, 4H, Ar–H), 7.56–8.17 (m, 3H, Benzothiazole-H), 8.30 (s, 1H, CH=N), 9.20 (s, 1H, CONH, D_2_O exchangeable); ^13^C NMR (CDCl_3_) *δ* ppm: 32.9, 46.2, 116.0, 118.2, 121.5, 123.7, 125.9, 129.2, 130.1, 132.8, 143.9, 147.9, 173.9, 174.5; MS (70 ev), *m*/*z*: 375.03 [M+H]^+^; Anal. Calcd. for C_17_H_15_ClN_4_O_2_S: C, 54.50, H, 4.09, N, 14.96, S, 8.55. Found: C, 54.53, H, 4.00, N, 14.97, S, 8.58.

*N-(6-Fluorobenzo[d]thiazol-2-yl)-3-[2-(2-hydroxybenzylidene)hydrazinyl]-propanamides (* ***5f*** *).* Yield 69%; IR (KBr) *ν* cm^−1^: 3350 (N–H str.), 1665 (C=O), 1615 (CH=N); ^1^H NMR (DMSO-*d*_6_) *δ* ppm: 2.65 (t, 2H, CH_2_), 2.90 (t, 2H,  CH_2_NH), 5.35 (s, 1H, OH), 6.10 (br s, 1H, NH, D_2_O exchangeable), 6.82–7.45 (m, 4H, Ar–H), 7.26–8.10 (m, 3H, Benzothiazole-H), 8.30 (s, IH, CH=N), 9.15 (s, 1H, CONH, D_2_O exchangeable); ^13^C NMR (CDCl_3_) *δ* ppm: 32.9, 46.2, 108.0, 113.2, 116.5, 121.5, 123.7, 126.2, 130.9, 132.9, 143.9, 144.9, 158.1, 173.9, 174.5; MS (70 ev), *m*/*z*: 360.09 [M+H]^+^; Anal. Calcd. for C_17_H_15_FN_4_O_2_S: C, 56.97, H, 4.29, N, 15.63, S, 8.96. Found: C, 56.99, H, 4.26, N, 15.60, S, 8.90.

*3-[2-(2-Hydroxybenzylidene)hydrazinyl]-N-(6-methylbenzo[d]thiazol-2-yl)propanamides (* ***5g*** *).* Yield 62%; IR (KBr) *ν* cm^−1^: 3346 (N–H str.), 1669 (C=O), 1585 (CH=N); ^1^H NMR (DMSO-*d*_6_) *δ* ppm: 2.35 (s, 3H, CH_3_), 2.66 (t, 2H, CH_2_), 2.93 (t, 2H,  CH_2_NH), 5.39 (s, 1H, OH), 5.95 (br s, 1H, NH, D_2_O exchangeable), 6.81–7.40 (m, 4H, Ar–H), 7.26–8.12 (m, 3H, Benzothiazole-H), 8.35 (s, 1H, CH=N), 10.27 (s, 1H, CONH, D_2_O exchangeable); ^13^C NMR (CDCl_3_) *δ* ppm: 24.0, 32.7, 46.2, 116.0, 118.9, 121.5, 121.7, 124.4, 126.0, 130.5, 132.0, 134.9, 143.9, 148.1, 173.7, 174.5; MS (70 ev), *m*/*z*: 358.12 [M+H]^+^; Anal. Calcd. for C_18_H_18_N_4_O_2_S: C, 62.00, H, 5.20, N, 15.90, S, 9.04. Found: C, 62.09, H, 5.24, N, 15.97, S, 9.03.

*3-[2-(2-Hydroxybenzylidene)hydrazinyl]-N-(6-methoxybenzo[d]thiazol-2-yl)propanamides (* ***5h*** *).* Yield 60%; IR (KBr) *ν* cm^−1^: 3320 (N–H str.), 1640 (C=O), 1600 (CH=N); ^1^H NMR (DMSO-*d*_6_) *δ* ppm: 2.60 (t, 2H, CH_2_), 2.89 (t, 2H,  CH_2_NH), 3.50 (s, 3H, OCH_3_), 5.40 (s, 1H, OH), 5.90 (br s, 1H, NH, D_2_O exchangeable), 6.81–7.40 (m, 4H, Ar–H), 7.06–8.12 (m, 3H, Benzothiazole-H), 8.36 (s, 1H, CH=N), 9.20 (s, 1H, CONH, D_2_O exchangeable); ^13^C NMR (CDCl_3_) *δ* ppm: 32.8, 46.2, 55.8, 105.8, 113.7, 116.0, 121.5, 122.8, 128.9, 130.1, 132.8, 141.8, 143.8, 156.8, 173.7, 174.5; MS (70 ev), *m*/*z*: 372.01 [M+H]^+^; Anal. Calcd. for C_18_H_18_N_4_O_3_S: C, 59.98, H, 4.84, N, 15.79, S, 8.86. Found: C, 60.01, H, 4.88, N, 15.85, S, 8.84.

*N-(6-Chlorobenzo[d]thiazol-2-yl)-3-[2-(4-hydroxybenzylidene)hydrazinyl]-propanamides (* ***5i*** *).* Yield 67%; IR (KBr) *ν* cm^−1^: 3355 (N–H str.), 1669 (C=O), 1590 (CH=N); ^1^H NMR (DMSO-*d*_6_) *δ* ppm: 2.63 (t, 2H, CH_2_), 2.91 (t, 2H,  CH_2_NH), 5.35 (s, 1H, OH), 6.10 (br s, 1H, NH, D_2_O exchangeable), 6.82–7.40 (m, 4H, Ar–H), 7.56–8.13 (m, 3H, Benzothiazole-H), 8.40 (s, 1H, CH=N), 10.09 (s, 1H, CONH, D_2_O exchangeable); ^13^C NMR (CDCl_3_) *δ* ppm: 32.9, 46.2, 116.0, 121.5, 123.9, 125.8, 126.0, 129.9, 130.6, 143.6, 147.8, 174.0, 175.8; MS (70 ev), *m*/*z*: 374.09 [M+H]^+^; Anal. Calcd. for C_17_H_15_ClN_4_O_2_S: C, 54.48, H, 4.09, N, 14.96, S, 8.75. Found: C, 54.50, H, 4.10, N, 14.98, S, 8.78.

*N-(6-Fluorobenzo[d]thiazol-2-yl)-3-[2-(4-hydroxybenzylidene)hydrazinyl]-propanamides (* ***5j*** *).* Yield 66%; IR (KBr) *ν* cm^−1^: 3349 (N–H str.), 1660 (C=O), 1610 (CH=N); ^1^H NMR (DMSO-*d*_6_) *δ* ppm: 2.60 (t, 2H, CH_2_), 2.95 (t, 2H,  CH_2_NH), 5.55 (s, 1H, OH), 5.82 (br s, 1H, NH, D_2_O exchangeable), 6.82–7.40 (m, 4H, Ar–H), 7.26–8.21 (m, 3H, Benzothiazole-H), 9.15 (s, 1H, CONH, D_2_O exchangeable); ^13^C NMR (CDCl_3_) *δ* ppm: 32.9, 46.2, 108.1, 113.7, 116.5, 123.7, 126.8, 130.6, 143.3, 144.6, 158.6, 160.6, 173.7, 175.8; MS (70 ev), *m*/*z*: 356.9 [M+H]^+^; Anal. Calcd. for C_17_H_15_FN_4_O_2_S: C, 56.89, H, 4.28, N, 15.76, S, 8.90. Found: C, 56.91, H, 4.23, N, 15.70, S, 8.93.

*3-[2-(4-Hydroxybenzylidene)hydrazinyl]-N-(6-methylbenzo[d]thiazol-2-yl)propanamides (* ***5k*** *).* Yield 66%; (KBr) *ν* cm^−1^: 3345 (N–H str.), 1666 (C=O), 1586 (CH=N); ^1^H NMR (DMSO-*d*_6_) *δ* ppm: 2.28 (s, 3H, CH_3_), 2.59 (t, 2H, CH_2_), 2.90 (t, 2H,  CH_2_NH), 5.28 (s, 1H, OH), 6.20 (br s, 1H, NH, D_2_O exchangeable), 6.81–7.40 (m, 4H, Ar–H), 7.36–8.41 (m, 3H, Benzothiazole-H), 8.52 (s, 1H, CH=N), 10.02 (s, 1H, CONH, D_2_O exchangeable); ^13^C NMR (CDCl_3_) *δ* ppm: 23.7, 32.5, 46.9, 116.0, 121.7, 123.8, 126.4, 130.6, 134.3, 143.6, 146.0, 173.7, 175.8; MS (70 ev), *m*/*z*: 356.12 [M+H]^+^; Anal. Calcd. for C_18_H_18_N_4_O_2_S: C, 61.01, H, 5.13, N, 15.90, S, 9.05. Found: C, 59.88, H, 5.20, N, 15.99, S, 8.99.

*3-[2-(4-Hydroxybenzylidene)hydrazinyl]-N-(6-methoxybenzo[d]thiazol-2-yl)propanamides (* ***5l*** *).* Yield 76%; IR (KBr) *ν* cm^−1^: 3348 (N–H str.), 1669 (C=O), 1600 (CH=N); ^1^H NMR (DMSO-*d*_6_) *δ* ppm: 2.55 (t, 2H, CH_2_), 2.98 (t, 2H,  CH_2_NH), 3.50 (s, 3H, OCH_3_), 5.32 (s, 1H, OH), 6.18 (br s, 1H, NH, D_2_O exchangeable), 6.79–7.40 (m, 4H, Ar–H), 7.29–8.45 (m, 3H, Benzothiazole-H), 8.49 (s, 1H, CH=N), 10.24 (s, 1H, CONH, D_2_O exchangeable); ^13^C NMR (CDCl_3_) *δ* ppm: 32.9, 46.2, 55.9, 105.0, 113.2, 116.0, 122.5, 125.8, 126.9, 141.8, 143.8, 156.9, 160.1, 173.7, 174.5; MS (70 ev), *m*/*z*: 370.11 [M+H]^+^; Anal. Calcd. for C_18_H_18_N_4_O_3_S: C, 58.40, H, 4.90, N, 15.12, S, 8.69. Found: C, 58.38, H, 4.88, N, 15.15, S, 8.72.

*N-(6-Chlorobenzo[d]thiazol-2-yl)-3-[2-(4-methylbenzylidene)hydrazinyl]-propanamides (* ***5m*** *).* Yield 72%; IR (KBr) *ν* cm^−1^: 3352 (N–H str.), 1671 (C=O), 1595 (CH=N); ^1^H NMR (DMSO-*d*_6_) *δ* ppm: 2.30 (s, 3H, CH_3_), 2.60 (t, 2H, CH_2_), 2.90 (t, 2H,  CH_2_NH), 5.80 (br s, 1H, NH, D_2_O exchangeable), 6.80–7.42 (m, 4H, Ar–H), 7.56–8.75 (m, 3H, Benzothiazole-H), 8.44 (s, 1H, CH=N), 9.15 (s, 1H, CONH, D_2_O exchangeable); ^13^C NMR (CDCl_3_) *δ* ppm: 24.3, 32.5, 46.0, 121.3, 123.8, 125.4, 129.1, 129.3, 140.6, 143.0, 147.7, 173.9, 174.0; MS (70 ev), *m*/*z*: 389.10 [M+H]^+^; Anal. Calcd. for C_18_H_17_ClN_4_OS: C, 57.69, H, 4.67, N, 15.07, S, 8.60. Found: C, 57.60, H, 4.60, N, 15.17, S, 8.54.

*N-(6-Fluorobenzo[d]thiazol-2-yl)-3-[2-(4-methylbenzylidene)hydrazinyl]-propanamides (* ***5n*** *).* Yield 77%; IR (KBr) *ν* cm^−1^: 3354 (N–H str.), 1669 (C=O), 1588 (CH=N); ^1^H NMR (DMSO-*d*_6_) *δ* ppm: 2.35 (s, 3H, CH_3_), 2.64 (t, 2H, CH_2_), 2.92 (t, 2H,  CH_2_NH), 6.20 (br s, 1H, NH, D_2_O exchangeable), 6.86–7.47 (m, 4H, Ar–H), 7.26–8.47 (m, 3H, Benzothiazole-H), 8.28 (s, 1H, CH=N), 10.27 (s, 1H, CONH, D_2_O exchangeable); ^13^C NMR (CDCl_3_) *δ* ppm: 24.3, 32.9, 46.2, 108.3, 113.8, 123.4, 126.1, 129.1, 129.2, 130.6, 143.0, 144.7, 158.6, 173.8, 175.0; MS (70 ev), *m*/*z*: 373.01 [M+H]^+^; Anal. Calcd. for C_18_H_17_FN_4_OS: C, 61.01, H, 4.87, N, 15.65, S, 8.89. Found: C, 59.01, H, 4.90, N, 15.52, S, 8.90.

*N-(6-Methylbenzo[d]thiazol-2-yl)3-[2-(4-methylbenzylidene)hydrazinyl] propanamides (* ***5o*** *).* Yield 80%; IR (KBr) *ν* cm^−1^: 3355 (N–H str.), 1670 (C=O), 1605 (CH=N); ^1^H NMR (DMSO-*d*_6_) *δ* ppm: 2.30 (s, 6H, 2CH_3_), 2.60 (t, 2H, CH_2_), 2.85 (t, 2H,  CH_2_NH), 5.88 (br s, 1H, NH, D_2_O exchangeable), 7.10–7.50 (m, 4H, Ar–H), 7.35–8.11 (m, 3H, Benzothiazole-H), 8.55 (s, 1H, CH=N), 10.22 (s, 1H, CONH, D_2_O exchangeable); ^13^C NMR (CDCl_3_) *δ* ppm: 23.6, 24.8, 32.1, 46.5, 121.4, 124.1, 126.1, 129.0, 129.2, 130.6, 134.0, 140.7, 146.6, 173.7, 174.9, MS (70 ev), *m*/*z*: 356.01 [M+H]^+^; Anal. Calcd. for C_19_H_20_N_4_O_2_S: C, 59.76, H, 4.81, N, 15.72, S, 9.00. Found: C, 59.79, H, 4.87, N, 15.68, S, 8.90.

*N-(6-Methoxybenzo[d]thiazol-2-yl)-3-[2-(4-methylbenzylidene)hydrazinyl]-propanamides (* ***5p*** *).* Yield 79%; IR (KBr) *ν* cm^−1^: 3316 (N–H str.), 1747 (C=O), 1610 (CH=N); ^1^H NMR (DMSO-*d*_6_) *δ* ppm: 2.34 (s, 3H, CH_3_), 2.62 (t, 2H, CH_2_), 2.92 (t, 2H,  CH_2_NH), 3.49 (s, 3H, OCH_3_), 5.92 (br s, 1H, NH, D_2_O exchangeable), 6.99–7.44 (m, 4H, Ar–H), 7.23–8.40 (m, 3H, Benzothiazole-H), 8.49 (s, 1H, CH=N), 10.28 (s, 1H, CONH, D_2_O exchangeable); ^13^C NMR (CDCl_3_) *δ* ppm: 21.5, 32.8, 46.5, 55.5, 104.9, 114.0, 118.0, 121.1, 123.0, 126.4, 129.9, 130.7, 143.6, 147.8, 163.0, 173.5, 175.8; MS (70 ev), *m*/*z*: 385.13 [M+H]^+^; Anal. Calcd. For C_19_H_20_N_4_O_3_S: C, 59.28, H, 5.26, N, 14.56, S, 8.49. Found: C, 59.62, H, 5.30, N, 14.66, S, 8.55.

*N-(6-Chlorobenzo[d]thiazol-2-yl)-3-[2-(4-methoxybenzylidene)hydrazinyl]-propanamides (* ***5q*** *).* Yield 82%, IR (KBr) *ν* cm^−1^: 3350 (N–H str.), 1660 (C=O), 1588 (CH=N); ^1^H NMR (DMSO-*d*_6_) *δ* ppm: 2.66 (t, 2H, CH_2_), 2.89 (t, 2H,  CH_2_NH), 3.49 (s, 3H, OCH_3_), 5.96 (br s, 1H, NH, D_2_O exchangeable), 7.10–7.44 (m, 4H, Ar–H), 7.15–8.40 (m, 3H, Benzothiazole-H), 8.35 (s, 1H, CH=N), 10.28 (s, 1H, CONH, D_2_O exchangeable); ^13^C NMR (CDCl_3_) *δ* ppm: 32.8, 46.5, 55.5, 114.0, 121.1, 123.0, 125.2, 126.4, 129.9, 130.7, 143.6, 147.8, 163.0, 173.5, 175.8; MS (70 ev), *m*/*z*: 388.09 [M+H]^+^; Anal. Calcd. for C_18_H_17_FCl_4_O_2_S: C, 56.59, H, 4.40, N, 15.11, S, 8.25. Found: C, 56.39, H, 4.35, N, 15.01, S, 8.29.

*N-(6-Fluorobenzo[d]thiazol-2-yl)-3-[2-(4-methoxybenzylidene)hydrazinyl]-propanamides (* ***5r*** *).* Yield 69%; IR (KBr) *ν* cm^−1^: 3353 (N–H str.), 1670 (C=O), 1599 (CH=N); ^1^H NMR (DMSO-*d*_6_) *δ* ppm: 2.63 (t, 2H, CH_2_), 2.96 (t, 2H,  CH_2_NH), 3.58 (s, 3H, OCH_3_), 6.05 (br s, 1H, NH, D_2_O exchangeable), 7.10–7.44 (m, 4H, Ar–H), 7.31–8.49 (m, 3H, Benzothiazole-H), 8.99 (s, 1H, CH=N), 10.19 (s, 1H, CONH, D_2_O exchangeable); ^13^C NMR (CDCl_3_) *δ* ppm: 32.6, 46.2, 56.5, 108.0, 113.1, 114.0, 123.2, 126.4, 129.9, 130.7, 143.6, 146.8, 158.0, 173.7, 174.8; MS (70 ev), *m*/*z*: 372.09 [M+H]^+^; Anal. Calcd. for C_18_H_17_FN_4_O_2_S: C, 58.05, H, 4.60, N, 15.04, S, 8.61. Found: C, 58.45, H, 4.68, N, 15.00, S, 8.69.

*3-[2-(4-Methoxybenzylidene)hydrazinyl]-N-(6-methylbenzo[d]thiazol-2-yl)propanamides (* ***5s*** *).* Yield 77%; IR (KBr) *ν* cm^−1^: 3354 (N–H str.), 1669 (C=O), 1579 (CH=N); ^1^H NMR (DMSO-*d*_6_) *δ* ppm: 2.35 (s, 3H, CH_3_), 2.58 (t, 2H, CH_2_), 2.92 (t, 2H,  CH_2_NH), 3.50 (s, 3H OCH_3_), 6.15 (br s, 1H, NH, D_2_O exchangeable), 6.86–7.47 (m, 4H, Ar–H), 7.26–8.47 (m, 3H, Benzothiazole-H), 8.44 (s, 1H, CH=N), 10.27 (s, 1H, CONH, D_2_O exchangeable); ^13^C NMR (CDCl_3_) *δ* ppm: 24.0, 32.9, 46.2, 55.5, 114.1, 121.2, 124.4, 126.9, 130.7, 143.9, 146.4, 163.0, 173.7, 174.5; MS (70 ev), *m*/*z*: 368.01 [M+H]^+^; Anal. Calcd. for C_19_H_20_N_4_O_2_S: C, 61.94, H, 5.45, N, 15.25, S, 8.71. Found: C, 61.99, H, 5.40, N, 15.30, S, 8.70.

*N-(6-Methoxybenzo[d]thiazol-2-yl)-3-[2-(4-methoxybenzylidene)hydrazinyl]-propanamides (* ***5t*** *).* Yield 69%; IR (KBr) *ν* cm^−1^: 3348 (N–H str.), 1669 (C=O), 1600 (CH=N); ^1^H NMR (DMSO-*d*_6_) *δ* ppm: 2.66 (t, 2H, CH_2_), 2.93 (t, 2H,  CH_2_NH), 3.59 (s, 6H, 2OCH_3_), 5.99 (br s, 1H, NH, D_2_O exchangeable), 6.79–7.44 (m, 4H, Ar–H), 7.29–8.45 (m, 3H, Benzothiazole-H), 8.36 (s, 1H, CH=N), 10.20 (s, 1H, CONH, D_2_O exchangeable); ^13^C NMR (CDCl_3_) *δ* ppm: 32.6, 46.0, 55.9, 105.1, 113.2, 114.4, 122.9, 125.7, 126.9, 130.4, 141.8, 143.0, 156.9, 160.2, 173.7, 174.5; MS (70 ev), *m*/*z*: 384.89 [M+H]^+^; Anal. Calcd. for C_19_H_20_N_4_O_3_S: C, 59.36, H, 5.24, N, 14.57, S, 8.34. Found: C, 59.39, H, 5.20, N, 14.59, S, 8.37.

### 2.2. Pharmacology

#### 2.2.1. Anticonvulsant Activity

The anticonvulsant activity was carried out on male albino mice (20–25 g) as experimental animals. The animals were housed under standard conditions and allowed free access to standard pellet diet and water. The pharmacological testing of all the final compounds was performed according to the standard protocol given by epilepsy branch of the National Institute of Neurological Disorders and Stroke (NINDS) following the protocol adopted by the Antiepileptic Drug Development (ADD) program. Phase I pharmacological screening comprised MES, scPTZ, and neurotoxicity. Compounds were administered intraperitoneally as a solution in polyethylene glycol (PEG). The most active compounds were evaluated quantitatively in phase II screening in which the ED_50_ and TD_50_ of the compounds were determined. These compounds were also tested for their median hypnotic dose (HD_50_) and median lethal dose (LD_50_) in phase III screening. To compare the bioavailability of the active compounds, the ED_50_ and TD_50_ values of the synthesized compounds were also determined after oral administration in phase IV screening.

*(1) Maximum Electroshock (MES) Test [17].* The compounds were screened for their anticonvulsant activity by electroshock seizure method. Seizures were elicited with a 60 Hz alternating current of 50 mA intensity in mice. The current was applied via ear-clip electrodes for 0.2 s. After i.p. administration of the compounds, the activities were evaluated at two time intervals, 0.5 h and 4 h. Protection against the spread of MES induced seizures was defined as the abolition of the hind limb and tonic maximal extension component of the seizure.

*(2) Subcutaneous Pentylenetetrazole (scPTZ) Seizure Threshold Test [18].* The subcutaneous dose of pentylenetetrazole (85 mg/kg) at which 95% of the animals showed convulsive reaction was determined by a dose-percent effect curve. The synthesized compounds were administered at the three graded doses, namely, 30, 100, and 300 mg/Kg, intraperitoneally. At the anticipated time, PTZ was then administered subcutaneously in the posterior midline of mice. Absence of clonic spasm in half or more of the animals in the observed time periods indicates the compounds capacity to terminate the effect of pentylenetetrazole on seizure threshold.

#### 2.2.2. Neurotoxicity Screening [19]

This test was performed using the rotarod method. At 30 min after the administration of the compounds, the animals were tested on a knurled plastic rod of diameter 3.2 cm rotating at 10 rpm for 1 min. Neurotoxicity was indicated by the inability of an animal to maintain equilibrium in each of three trials.

#### 2.2.3. Liver Function Test [20–23]

To find out the toxic effects, if any, of the synthesized compounds on liver, the test compounds were administered to mice. After 24 hours, serum samples were taken for estimation of serum glutamate oxaloacetate transaminase (SGOT), serum glutamate pyruvate transaminase (SGPT), alkaline phosphatase (ALP), albumin, and bilirubin using commercially available kits.

## 3. Result and Discussion

### 3.1. Chemistry

**Scheme 1:**
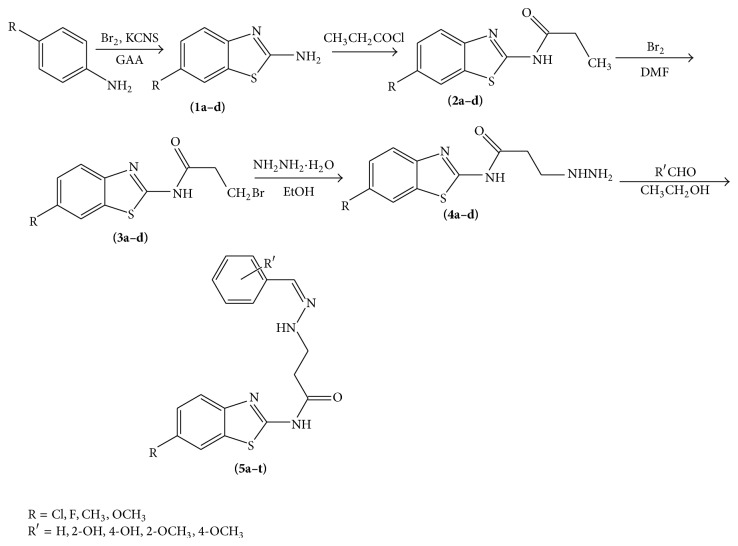
Synthetic route to the titled compounds (**5a**–**t**).

A series of new benzothiazole derivatives were synthesized in satisfactory yields (65–80%) as demonstrated in Scheme 1 and their structures were characterized by elemental and spectral analysis. The physicochemical properties of synthesized compounds are presented in Table 1. Substituted anilines are cyclised to 6-substituted benzo*[d]*thiazole-2-amines (**1a**–**d**) on treatment with potassium thiocyanates, bromine in glacial acetic acid, which, on treatment with propionyl chloride, afforded N-(6-substituted benzo*[d]*thiazol-2-yl)propanamides (**2a**–**d**). This compound on bromination yields 3-bromo-*N*-(6-substituted benzo*[d]*thiazol-2-yl)propanamides (**3a**–**d**). On further treatment with hydrazine, hydrate converted into* N*-(6-substituted benzo*[d]*thiazol-2-yl)-3-hydrazinylpropanamide (**4a**–**d**), which was condensed with different aldehydes to yield the titled compounds 3-[2-(substituted benzylidene)]-*N*-(6-substituted benzo*[d]*thiazol-2-yl)propanamides (**5a**–**t**).

The synthesized benzothiazole derivatives showed (N–H), (C=O), and (C=N) stretching bands in the region of 3355–3316 cm^−1^, 1747–1640 cm^−1^, and 1615–1569 cm^−1^, respectively, in their IR spectrum, while, in their ^1^H NMR spectra, these compounds exhibited multiplets for (Ar–H) in the regions of *δ* 6.80–7.80 ppm, a singlet in the region of *δ* 9.15–10.30 ppm (CONH), and a singlet for (CH=N) in the region of *δ* 8.20–8.99 ppm. ^13^C spectra of prototype compound** 5p** showed peaks at *δ* 21.5, 32.8, 46.5, 55.5, 104.9, 114.0, 118.0, 121.1, 123.0, 126.4, 129.9, 130.7, 143.6, 147.8, 163.0, 173.5, and 175.8 ppm confirming presence of 17 different carbon atoms.

### 3.2. Pharmacology

A pragmatic approach to synthesize new series of benzothiazole derivatives in satisfactory yields was illustrated in Scheme 1. The result of anticonvulsant activity of the compounds (**5a**–**t**) is depicted in Table 2. Phase I preliminary anticonvulsant screening revealed that most of the newly synthesized compounds showed some degree of protection in MES screen, which suggested the good ability of these compounds to stop the seizure spread at a certain dose level. In the MES test, compounds** 5h** and** 5p** have shown protection against MES induced seizures at dose of 30 mg/kg after 0.5 h of administration. Fascinatingly, compound** 5p** exhibited continued protection against seizures at the same dose after 4.0 h also. It signified that compound** 5p** has rapid onset and long duration of anticonvulsant activity at lower dose and the result is comparable with the standard drug, phenytoin. Compounds that were active at a dose of 100 mg/kg after 0.5 h in MES screen included** 5a**,** 5d**,** 5f**,** 5i**,** 5k**,** 5n**,** 5o**,** 5s**, and** 5t** representing that they have rapid onset and short duration of anticonvulsant activity.

The synthesized compounds challenged the scPTZ test to predict their potential against seizure threshold. Compounds** 5a**,** 5c**,** 5h**,** 5i**, and** 5p** were active against seizures after 0.5 h at a dose of 100 mg/kg. Compounds** 5d**,** 5e**,** 5f**,** 5j**,** 5m**,** 5n**,** 5r**, and** 5t** were active at 300 mg/kg after the same time period. Only compounds** 5a**,** 5i**,** 5j**, and** 5t** were active after 4.0 h at a dose of 300 mg/kg indicative of the long duration of action of these compounds at high dose.

In the neurotoxicity screen, most of the compounds did not show any neurotoxicity. Compounds** 5i**,** 5o**, and** 5r** revealed neurotoxicity at a dose of 300 mg/kg after 0.5 h and compounds** 5b**,** 5j**,** 5n**, and** 5p** exhibited neurotoxicity after 4.0 h.

In phase II anticonvulsant screening, the most active compounds** 5h** and** 5p** exhibited, in the MES screen, ED_50_ of 27.9 mg/kg and 28.4 mg/kg, respectively, TD_50_ of 378.5 mg/kg and 287.1 mg/kg, respectively, and protective index (PI) of 13.5 and 10.1, respectively, which is higher as compared to phenytoin and carbamazepine. In the scPTZ screen,** 5h** and** 5p** offered protection with an ED_50_ of 188.6 mg/kg and 89.1 mg/kg, respectively, a PI of 2.0 and 3.2, respectively, higher than standard drugs (Table 3). The protective index showed significant results. Higher PI values in MES and scPTZ screen indicated that compounds** 5h** and** 5p** are safer and effective anticonvulsant agents. Since both the compounds have shown potential in both phase I and phase II screening, they were subjected to be further assessed in phase III and phase IV screening.

In phase III screening, the toxicity profile of compounds** 5h** and** 5p** was determined and the results are revealed in Table 4. Mice were injected i.p. with the test compounds at different doses in order to determine the HD_50_ for the hypnotic activity of the compounds based on loss of the righting reflex. Groups of 10 animals were used for each dose. Solutions were prepared immediately before the test. Logarithmic dose-response curves for test compounds were fitted to calculate the HD_50_ using a linear regression analysis. Data are reported as means ± SE. For LD_50_, the selected compounds were administered intraperitoneally to mice at various doses in the multiple of TD_50_ and the toxicity persuaded by them was portrayed by diminished motor activity, relaxation of muscles, failure of righting reflex, and decline level of respiration. At higher doses, animals also showed hypnosis, analgesia, and anesthesia. The median hypnotic dose (HD_50_) of compound** 5h** was found to be 642 mg/kg, which is nearly twice the TD_50_ of the compound. It also showed the 24 h median lethal dose (LD_50_) of 865 mg/kg. Compound** 5p** displayed the HD_50_ value 712 mg/kg with LD_50_ 650 mg/kg. Both compounds showed high safety profile as the HD_50_/ED_50_ values of** 5h** and** 5p** were found to be 23.01 and 25.07 against MES induced seizures and 3.4 and 8.0 against scPTZ induced seizures. These values are higher than that showed by phenytoin. They displayed a considerable safety profile in scPTZ induced seizure also indicative of the efficiency of both the compounds as broad spectrum anticonvulsants.

In phase IV anticonvulsant screening, the selected compounds** 5h** and** 5p** were further evaluated for ED_50_ and TD_50_ values after oral administration in mice to assess their bioavailability. The result indicated that, on oral administration, the bioavailability of test compounds decreased since the ED_50_ values were found to be higher than the ED_50_ values in phase II screening on i.p. administration (Table 5).

**Figure 1:**
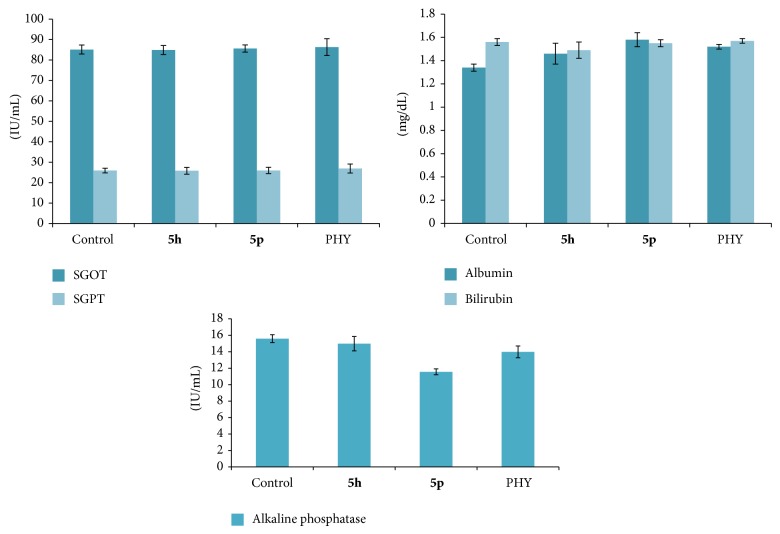
Hepatic enzymes estimations after treatment with different test compounds. Number of animals tested (*n* = 6). The mean levels were calculated using ANOVA followed by Dunnett's test.

**Figure 2:**
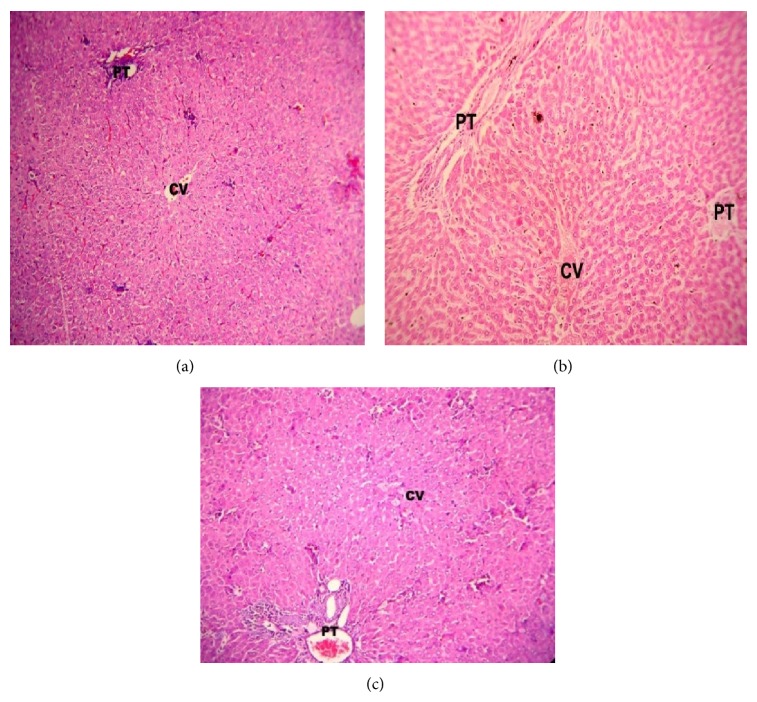
High power photomicrograph of portal triad area of liver tissue from animals treated with (a) control, (b) compound (**5h**), and (c) compound (**5p**) showing a normal histological appearance (HE ×400). PT, portal triad; CV, central vein.

Phenytoin is a probable cause of acetaminophen hepatotoxicity [24] and anticonvulsants such as carbamazepine [25] and valproic acid [26] were also expected to enhance or show hepatotoxicity as a major side effect. Selected active compounds** 5h** and** 5p** were investigated for their hepatotoxic side effects by means of liver function tests (Table 6). Compounds were administered chronically to mice for 2 weeks and the biochemical parameters were estimated. The values of alkaline phosphatase, serum glutamate oxaloacetate transaminase (SGOT), serum glutamate pyruvate transaminase (SGPT), albumin, and bilirubin suggested that none of the compounds had shown any considerable increase or decrease (Figure 1). Further histopathological study of compounds** 5h** and** 5p** confirmed that there is no liver toxicity by showing normal hepatic parenchyma with portal triad, central vein, and hepatocytes in comparison to control (Figure 2).

## 4. Conclusion

In the present study, a series of 3-[2-(2-substituted benzylidene)hydrazinyl]-*N*-(6-substitutedbenzo*[d]*thiazol-2-yl)propanamides were synthesized successfully and all compounds were tested for anticonvulsant activity (phases I–IV) using MES and scPTZ screens. Compounds** 5h** and** 5p** represent valuable leads in the exploration of agents controlling both treatment of seizures and intoxication during epilepsy. Hence, we may conclude that reported series of substituted benzothiazole derivatives may be promising for the development of potential anticonvulsant agents after further optimization.

## Figures and Tables

**Table 1:** Physicochemical parameters of the synthesized compounds (**5a**–**t**).

Compound	R	R′	Molecular formula	Log ⁡*P* ^a^	*C*Log*P* ^b^	M.P (°C)	*R*_*f*_^c^
**5a**	Cl	H	C_17_H_15_ClN_4_OS	4.32	4.14	192–194	0.42
**5b**	F	H	C_17_H_15_FN_4_OS	3.92	3.57	188–190	0.35
**5c**	CH_3_	H	C_18_H_18_N_4_OS	4.24	3.92	130–132	0.81
**5d**	OCH_3_	H	C_18_H_18_N_4_O_2_S	3.63	3.72	202–204	0.32
**5e**	Cl	2-OH	C_17_H_15_ClN_4_O_2_S	3.93	4.73	198–200	0.59
**5f**	F	2-OH	C_17_H_15_FN_4_O_2_S	3.53	3.53	164–166	0.44
**5g**	CH_3_	2-OH	C_18_H_18_N_4_O_2_S	3.86	4.46	180–182	0.61
**5h**	OCH_3_	2-OH	C_18_H_18_N_4_O_3_S	3.24	4.32	110–112	0.78
**5i**	Cl	4-OH	C_17_H_15_ClN_4_O_2_S	3.95	4.76	210–212	0.31
**5j**	F	4-OH	C_17_H_15_FN_4_O_2_S	3.59	3.61	201–203	0.48
**5k**	CH_3_	4-OH	C_18_H_18_N_4_O_2_S	3.89	3.97	185–187	0.62
**5l**	OCH_3_	4-OH	C_18_H_18_N_4_O_3_S	3.24	4.32	117–119	0.57
**5m**	Cl	4-CH_3_	C_18_H_17_ClN_4_OS	4.8	4.61	175-176	0.65
**5n**	F	4-CH_3_	C_18_H_17_FN_4_OS	4.44	4.06	190–192	0.4
**5o**	CH_3_	4-CH_3_	C_19_H_20_N_4_OS	4.73	4.44	205-206	0.71
**5p**	OCH_3_	4-CH_3_	C_19_H_20_N_4_O_2_S	4.12	4.22	200-201	0.6
**5q**	Cl	4-OCH_3_	C_18_H_17_ClN_4_O_2_S	4.19	4.42	140–142	0.55
**5r**	F	4-OCH_3_	C_18_H_17_FN_4_O_2_S	3.79	3.85	169–171	0.43
**5s**	CH_3_	4-OCH_3_	C_19_H_20_N_4_O_2_S	4.12	4.20	161–163	0.38
**5t**	OCH_3_	4-OCH_3_	C_19_H_20_N_4_O_3_S	4.75	4.41	215–217	0.54

Solvent of crystallization-ethanol. ^a^Log⁡ *P* was calculated using octanol-phosphate buffer. ^b^ *C*Log*P* was calculated using software chem. Draw ultra 8. ^c^Solvent system-toluene : ethyl acetate : formic acid (5 : 4 : 1), benzene : acetone (8 : 2).

**Table 2:** Phase I anticonvulsant evaluation of the synthesized compounds (**5a–t**).

Compound	Intraperitoneal injection in mice					
	MES		scPTZ		Neurotoxicity screen	
	0.5 h	4.0 h	0.5 h	4.0 h	0.5 h	4.0 h
**5a**	100	100	100	300	—	—
**5b**	300	—	—	—	—	300
**5c**	—	300	100	—	—	—
**5d**	100	300	300	—	—	—
**5e**	300	—	300	—	—	—
**5f**	100	300	300	—	—	—
**5g**	300	—	—	—	—	—
**5h** ^*^	30	100	100	—	—	—
**5i**	100	—	100	300	300	—
**5j**	300	—	300	300	—	300
**5k**	100	300	—	—	—	—
**5l**	—	300	—	—	—	—
**5m**	—	—	300	—	—	—
**5n**	100	—	300	—	—	300
**5o**	100	300	—	—	300	—
**5p** ^*^	30	30	100	—	—	300
**5q**	300	300	—	—	—	—
**5r**	300	—	300	—	300	—
**5s**	100	300	—	—	—	—
**5t**	100	—	300	300	—	—
PHY	30	30	—	—	100	100
CBZ	30	100	100	300	100	300

Number of animals in each group (*n*) = 4; solvent used-polyethylene glycol; doses of 30, 100, and 300 mg/kg were administered i.p. The figures indicate the minimum dose whereby bioactivity was demonstrated in half or more mice. The (—) indicates an absence of activity at maximum dose administered (300 mg/kg).

**Table 3:** Phase II quantitative anticonvulsant evaluation of selected active compounds.

Compound	ED_50_^a^		TD_50_^b^	PI^c^	
	MES	scPTZ		MES	ScPTZ
**5h**	27.9 ± 1.43^d^	188.6 ± 9.23	378.5 ± 17.09	13.5	2.0
**5p**	28.4 ± 0.88	89.1 ± 7.72	287.1 ± 22.13	10.1	3.2
PHY	9.5 ± 0.77	>300	65.5 ± 12.06	6.9	<0.22
CBZ	15.8 ± 1.02	>100	71.6 ± 12.07	8.1	<0.22

Number of animals used = 08; solvent used: polyethylene glycol (0.1 mL, i.p.), ^a^ED_50_—median effective dose eliciting anticonvulsant protection in 50% animals.

^b^TD_50_ median toxic dose eliciting minimal neurological toxicity in 50% animals.

^c^PI = protective index (TD_50_/ED_50_). ^d^Data in parentheses are the 95% confidence limits.

**Table 4:** Phase III quantitative toxicity profile of selected compounds.

Compound	HD_50_^a^	LD_50_^b^	HD_50_/ED_50_	
			MES	PTZ
**5h**	642.2 (609.7–689.6)^c^	865.9 (821.1–902.7)	23.01	3.41
**5p**	712.3 (664.9–768.2)	650.5 (609.1–697.5)	25.07	8.00
PHY	182.4 (169.3–94.2)	224.8 (201.3–249.2)	19.15	>0.06

^a^Median hypnotic dose in mg/kg; ^b^median lethal dose in mg/kg; mortality was determined 24 h after i.p. injection.

^c^95% confidence interval in parenthesis.

**Table 5:** Phase IV quantitative anticonvulsant evaluation of selected active compounds after oral administration.

Compound	TPE^a^ (h)	ED_50_^b^ (mg/kg)	TD_50_^c^ (mg/kg)	PI^d^
**5h**	2	39.8 (33.1–44.5)^e^	456.3 (419.2–502.2)	11.4
**5p**	2	48.5 (41.3–54.6)	689.1 (643.8–720.4)	14.2
PHY	2	9.16 (7.9–11.4)	87.6 (78.3–98.4)	9.56

^a^Time of peak pharmacodynamic activity.

^b^ED_50_ median effective dose eliciting anticonvulsant protection in 50% animals.

^c^TD_50_ median toxic dose eliciting minimal neurological toxicity in 50% animals.

^d^PI (protective index) was determined by TD_50_/ED_50_.

^e^95% confidence interval in parenthesis.

**Table 6:** Enzyme estimation of the selected compounds.

Compound	SGOT^a^ level (IU/mL)	SGPT^b^ level (IU/mL)	Alkaline phosphatase (IU/mL)	Albumin (mg/dL)	Bilirubin (mg/dL)
Control	**85.1** ± 2.18^*^	**25.91** ± **1.13**	**15.59** ± **0.48**	**1.34** ± **0.03**	**1.56** ± **0.03**
**5h** ^*^	84.9 ± 2.21	25.81 ± 1.65	14.98 ± 0.87	1.46 ± 0.09	1.49 ± 0.07
**5p** ^*^	85.6 ± 1.76	25.94 ± 1.56	11.56 ± 0.36	1.58 ± 0.06	1.55 ± 0.03
PHY	86.3 ± 4.12	26.91 ± 2.20	13.98 ± 0.72	1.52 ± 0.02	1.57 ± 0.02

Number of animals tested (*n* = 6), ^a^serum glutamate oxaloacetate transaminase, ^b^serum glutamate pyruvate transaminase, ^*^ *P* > 0.05 versus control. The mean levels were calculated using ANOVA followed by Dunnett's.
